# Unacceptable use of substandard metrics in policy decisions which mandate large reductions in animal-source foods

**DOI:** 10.1038/s41538-024-00249-y

**Published:** 2024-02-05

**Authors:** Alice V. Stanton

**Affiliations:** School of Pharmacy and Biomolecular Sciences, Royal College of Surgeons in Ireland, University of Medicine and Health Sciences, Dublin, Ireland

**Keywords:** Risk factors, Cancer, Agriculture

## Abstract

Many recent very influential reports, including those from the Global Burden of Disease (GBD) Risk Factor Collaborators, the EAT-Lancet Commission on Food, Planet, Health, and the Lancet Countdown on Health and Climate Change, have recommended dramatic reductions or total exclusion of animal-source foods, particularly ruminant products (red meat and dairy), from the human diet. They strongly suggest that these dietary shifts will not only benefit planetary health but also human health. However, as detailed in this perspective, there are grounds for considerable concern in regard to the quality and transparency of the input data, the validity of the assumptions, and the appropriateness of the statistical modelling, used in the calculation of the global health estimates, which underpin the claimed human health benefits. The lessor bioavailability of protein and key micronutrients from plant-source foods *versus* animal-source foods was not adequately recognised nor addressed in any of these reports. Furthermore, assessments of bias and certainty were either limited or absent. Despite many of these errors and limitations being publically acknowledged by the GBD and the EAT-Lancet authors, no corrections have been applied to the published papers. As a consequence, these reports continue to erroneously influence food policy decisions and international dietary guidelines, such as the World Wildlife Fund’s Livewell Diet, and the Nordic Nutrition Recommendations 2023.

The world in 2023 faces climate and biodiversity crises. Food production and consumption contributes importantly to both of these crises. The food system is currently estimated to be responsible for about a third of total greenhouse gas emissions^1^, and the conversion of natural ecosystems to agricultural land has been reported to be the largest threat to species extinction^2^. Hence, there is indeed a need to transform our food system so that all have access to healthy diets, while at the same time safeguarding the planet’s health. The details of how that is best achieved is the subject of considerable debate – how much change should come from each section of the food system – how much change from food production, processing, distribution and retailing and how much from consumption?

Many recent publications have identified dietary shift as a key food system transformation^3–7^. Rather than recommending moderation of current consumption patterns, these papers require considerable reductions, or even total exclusion of animal-source foods, particularly ruminant products (red meat and dairy), from the human diet. They propose that these dramatic dietary shifts would benefit both planetary and human health. In this perspective, the reliability of the claims for benefits for human health is examined.

Reports from two groups, namely from the EAT-Lancet Commission on Food, Planet, Health^3^, and from the Global Burden of Diseases (GBD) Risk Factors Collaborators^8–12^, are examined in particular detail. This is because estimates and reports from these two groups are very influential. Indeed many other reports and policy papers cite evidence from these two groups, and/or use the same assumptions and analytical techniques.

The EAT-Lancet Commission on Food, Planet and Health published its first report in The Lancet in January 2019^3^. This paper, which described a planetary health diet designed to feed the world’s growing population without costing the Earth, made headlines across the world. On social media, content connected to the report have had more than one million shares in over 200 countries. According to Altmetric, the report is amongst the top 20 most discussed science papers across all academia, having been cited by 4542 scientific papers and 631 policy documents in the 4.5 years since publication

For the past 30 years, reports from the GBD Collaborators have been used by researchers, governments and non-governmental organisations to make comparisons amongst populations, to track changes over time, to monitor progress toward the Sustainable Development Goals, and to inform policy. Their outputs are widely cited in the scientific literature and in policy documents of the United Nations, the World Health Organisation, the European Commission, many national and international food systems strategies and dietary guidelines^13–16^. Reflecting on this influence, GBD leaders from the Institute of Health Metrics and Evaluation, University of Washington, Seattle, have described the GBD studies as “the de-facto source for global health accounting”^8^.

Because of data gaps and measurement challenges in nutritional science, most, if not all, of the reports proposing considerable reductions in animal-source foods have used global health estimates, rather than primary data, as evidence for their recommendations. Hence, in this perspective, the quality and transparency of the input data, the validity of the assumptions of the statistical models used in the calculation of the health estimates, and the conduct of post publication processes, are considered. The grounds for considerable concern are described in the following sections.

## Validity of assumptions - recommended optimal intakes of the GBD 2019 risk factors study and the EAT-Lancet planetary health diet

The Scientific Group of the UN Food Systems Summit 2021 defined a healthy diet as “health-promoting and disease-preventing” and as “providing adequacy without excess, of nutrients and health promoting substances from nutritious foods, and avoids the consumption of health-harming substances”^17^. The World Health Organisation similarly describes healthy diets as “helping to protect against malnutrition in all its forms, as well as noncommunicable diseases, including diabetes, heart disease, stroke and cancer.”^18^

In the GBD 2019 Risk Factors Study, for both red and processed meats, the level of exposure associated with the lowest level of risk, called the theoretical minimum risk exposure level (TMREL), was, by default, set to zero. This was very puzzling, as the contribution of moderate consumption of red and processed meats to nutrient adequacy, appeared to be totally ignored. Red and processed meats are rich in all essential amino acids and in many commonly lacking micronutrients, including iron, zinc, vitamin B_12_ and vitamin D_3_^19–21^. Whilst red and processed meats are not the only sources for these nutrients, for many populations worldwide, they are the most important bioavailable sources. If the current public health message advising moderate consumption of red and processed meats, as part of a healthy balanced diet, was to be replaced by GBD 2019 guidance that any intake of such meats was harmful, it is highly likely that the prevalence of child and maternal malnutrition, iron deficiency anaemia, and elderly sarcopenia, would be greatly increased. It is of importance that the GBD risk factor collaborators have responded to this issue, and confirmed that the TMREL for unprocessed red meat in future reports will not be zero^22,23^. Even more recently, they have stated that the 95% uncertainty interval for this estimate is very wide (0–200 g/day)^11^ – this indicates that the optimal intake of red meat could be as high as 200 g a day.

**Fig. 1:**
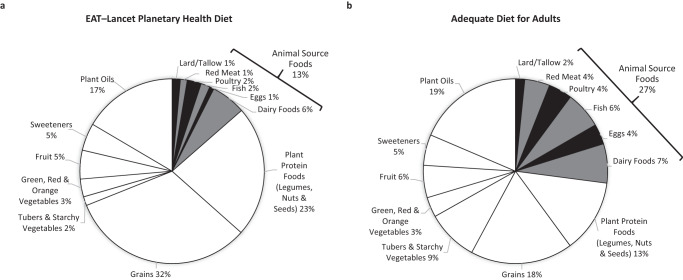
Quantities of foods recommended by the EAT-Lancet Planetary Health Diet and the Adequate Diet for Adults. Comparison of the percentages of calories provided by various food groups in the EAT-Lancet planetary health reference diet (panel **a**)^3^, and in a diet which provides sufficient micronutrients, the adequate diet for adults (panel **b**)^27^.

The EAT-Lancet planetary health reference diet does allow low quantities of red or processed meats and eggs to be consumed, and includes moderate amounts of seafood and poultry. However the diet largely consists of vegetables, fruits, whole grains, legumes, nuts and unsaturated plant oils, and only 13% of calories are from animal-source foods^3^ (Fig. 1a).

In 2019, the EAT-Lancet Commission were confident that this diet would meet all nutritional requirements of all adults and of children older than 2 years. However, others questioned whether the considerable limitation of animal-source foods in the diet would negatively impact on protein and micronutrient adequacy, particularly for women, children and the elderly, and would result in adverse consequences for developing and aging brains^24–26^. Hence, I welcome the recent acknowledgement, by at least some of the EAT-Lancet Commissioners, that this first version of the planetary health diet would indeed result in significant essential micronutrient shortfalls^27^. This particularly pertains to micronutrients found in higher quantities, and in more bioavailable forms, in animal-source foods, such as vitamin B_12_, calcium, iron and zinc. It appears that insufficient attention was paid to the latest evidence on recommended nutrient intakes, and to the differences in micronutrient bioavailability from plant-source foods *versus* animal-source foods^27,28^. In order to achieve micronutrient adequacy, intakes of animal-source foods would have to be doubled, accounting for at least 27% of calories, and intakes of phytates-rich plant-sourced foods, such as whole grains, pulses and nuts, would need to be considerably reduced (Fig. 1b)^27^.

In 2019, the EAT-Lancet Commission also expressed confidence that widespread uptake of their recommended diet would reduce the incidence of non-communicable diseases and overall mortality - they estimated that approximately 11 million premature deaths among adults could be avoided annually through global adoption of the diet^3^. Zagmutt and colleagues were the first to question these estimates of avoided mortalities – they identified flaws in the assumptions and methods used, and their corrected analysis suggested that any mortality reduction effect of the EAT-Lancet diet was no greater than the impact of energy consumption changes that would prevent under-weight, over-weight and obesity alone^29,30^. Adherence to the EAT-Lancet reference diet did show beneficial associations for ischaemic heart disease and diabetes mellitus, but not for stroke nor mortality in the EPIC-Oxford study^31^, and was associated with reduced risks of cancer and all-cause mortality, but no association with cardiovascular events, in the UK Biobank cohort^32^. The authors of both of these UK-based studies noted that those most adherent to the EAT-Lancet diet were also most likely to follow a healthy lifestyle, and therefore residual confounding might operate. Furthermore, close adherence to the planetary health diet provided no additional protection from major cardiovascular events, cancer or mortality, in either the Prospective NutriNet-Santé cohort^33^, or the Prospective Urban Rural Epidemiology study^34^.

## Quality and transparency of the input data - use of unpublished data by the GBD 2019 risk factors study

Compared with the GBD 2017 estimates^9^, the GBD 2019 estimates of the risks of many dietary factors^10^ differed considerably. Disease burdens (as measured by deaths and disability adjusted life-years [DALYs]) attributed to diets low in fruit, nuts and seeds, vegetables, seafood omega-3 fatty acids, polyunsaturated fatty acids, vitamin A or zinc declined by more than 50%, whereas risks for diets low in legumes, or high in either processed meats or trans fats, more than doubled. However, the most substantial change in the 2019 estimates was the disease burden attributed to diets high in unprocessed red meat. The GBD 2019 Risk Factor collaborators reported finding “sufficient evidence supporting a causal relationship of red meat intake with colorectal cancer, breast cancer, type 2 diabetes, ischaemic heart disease, ischaemic stroke and haemorrhagic stroke”. They estimated that 896,000 deaths (95% uncertainty interval, 536,000–1,250,000) and 23.9 million (15.6–32.0) disability-adjusted life years (DALYs) were attributable to unprocessed meat consumption globally in 2019. This represented 36-fold and 18-fold increases over the GBD 2017 estimates, respectively.

Whilst all previous GBD analyses, including the GBD 2017 analysis, used data from published systematic reviews and meta-analyses, the evidence for the 2019 dietary risk factor estimates came from in-house, newly conducted, systematic reviews and meta-regressions. These analyses had not been peer-reviewed nor published, and no assessments of certainty were documented. I and many scientists, including representatives of the Academy of Nutrition Sciences and the World Cancer Research Fund (WCRF), questioned the reliability of the dramatically changed 2019 estimates^22,35^, and requested that PRISMA (Preferred Reporting Items for Systematic Reviews and Meta-Analyses)^36^ compliant reports of the newly conducted systematic reviews be peer-reviewed and published.

It is good that these requests were eventually answered through publication of the Burden of Proof (BoP) study of the health effects associated with consumption of unprocessed red meat in Nature Medicine in October 2022^11^. However, this publication raises further issues.

**Fig. 2:**
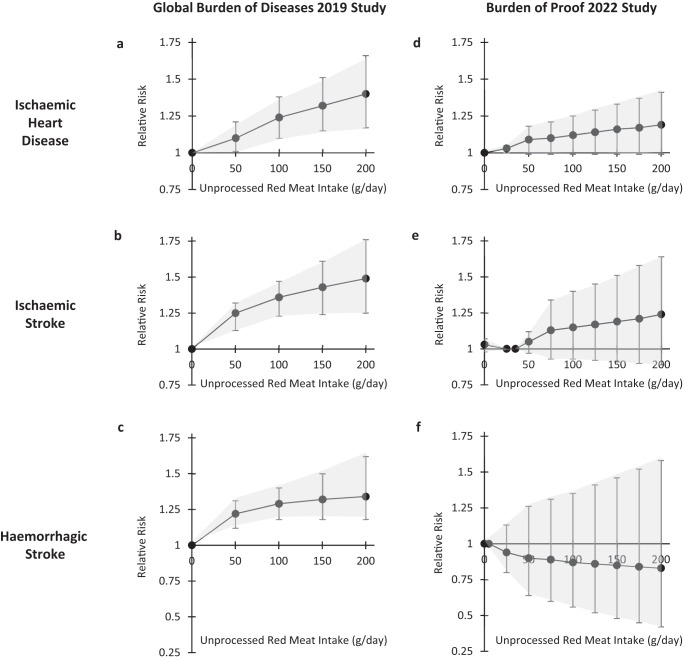
Associations between unprocessed red meat consumption and cardiovascular outcomes. Relative risk estimates from the GBD 2019 Risk Factors Study (panels **a**–**c**) and the Burden of Proof 2022 Study (panels **d**–**f**) for ischaemic heart disease, ischaemic stroke and haemorrhagic stroke, by levels of unprocessed red meat intake. Relative risks (95% confidence intervals) are plotted on the y-axis and unprocessed red meat intake (g/day) is plotted on the x-axis. The relative risk curves of the GBD 2019 Risk Factors Study (panels **a**–**c**) are for men and women aged 50–54 years, and were drawn using data from Table S7A of Supplementary Appendix 1^10^. The relative risk curves of the Burden of Proof 2022 Study (panels **d**–**f**) are for adult men and women, and were drawn using data from Supplementary Table 7^11^.

The relative risk curves and the conclusions of the BoP 2022 Study^11^ are very different from those reported in the GBD 2019 Risk Factors Study^10^. This is particularly striking for the three cardiovascular outcomes. The GBD 2019 Study indicated that risks for ischaemic heart disease, ischaemic stroke and haemorrhagic stroke increased significantly even with moderate intakes of red meat (50 g/day), and that risk increased further with greater intakes (Fig. 2a–c). By contrast, the relative risk curves of the BoP 2022 study are either considerably flatter (ischaemic heart disease and ischaemic stroke, Fig. 2d, e), or show a trend towards protection (haemorrhagic stroke, Fig. 2f). It is noteworthy that none of these BoP relative risk curves are statistically significant, even with intakes as high as 200 g/day. The overall conclusions of the BoP 2022 study were that there is no or only very weak evidence that unprocessed red meat is associated with any increased risk. These BoP findings are in agreement with other recently conducted systematic reviews of randomised trials and cohort studies^37–39^.

The large disparities between the conclusions of the GBD 2019 and BoP 2022 reports concerning unprocessed red meat, highlights the importance of expert peer review, of compliance with the PRISMA statement for all newly conducted systematic reviews and metanalyses^36^, and of compliance with the GATHER (Guidelines for Accurate and Transparent Health Estimates Reporting) statement for all reports of global health estimates^40^. The large disparities also cast considerable doubt over the accuracy of the GBD 2019 estimates of the risks attributed to all other dietary factors, given that these estimates are also based on systematic reviews and meta-regressions which have not been peer-reviewed nor published.

## Appropriateness of statistical modelling – monotonic constraints, risk of bias and certainty assessments in the burden of proof 2022 studies

As already mentioned, the GBD collaborators published a series of papers in Nature Medicine in October 2022^11,12,41,42^, which described and demonstrated their newly developed Burden of Proof methodology. Key steps of the novel modelling methodology include;

1. Estimation of the shape of the risk-outcome relationship using quadratic splines;
2. Application of monotonicity or linear-tail constraints;
3. Automatic detection and trimming of outliers;
4. Testing and correction for bias related to study design and reporting bias;
5. Computation of 95% uncertainty intervals which account for mean effects uncertainty and between-study heterogeneity;
6. Generation of the burden of proof risk function (BPRF), which estimates the level of risk closest to the null hypothesis, that is consistent with available data; and
7. Classification of each risk–outcome pair into five categories of certainty (star ratings of 1-5) based on the average magnitude of the BPRF.

Zheng and colleagues consider the existing mechanisms to quantify and rank the magnitude and uncertainty of health risks, such as the GRADE (Grading of Recommendations Assessment, Development and Evaluation) approach and Cochrane Reviews, to be largely subjective^12^. They propose that the Burden of Proof approach will provide a more consistent way to understand, evaluate and summarise evidence of risk across different risk-outcome pairs. They confirm that this methodology will inform the risk analyses of future GBD risk factor studies^12,41^. However, there appear to be a number of important flaws and limitations to this new methodology.

**Fig. 3:**
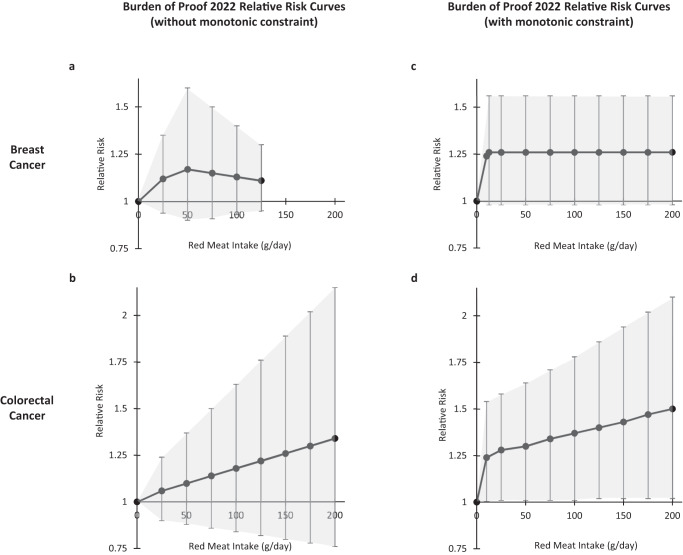
Risk inflationary effects of default application of monotonic constraints. Relative risk estimates from the Burden of Proof 2022 Study for breast and colorectal cancer, by levels of unprocessed red meat intake, both prior to (panels **a** and **b**), and after (panels **c** and **d**) application of a monotonic constraint^11^. Relative risks (95% confidence intervals) are plotted on the y-axis and unprocessed red meat intake (g/day) is plotted on the x-axis. The relative risk curves are for adult men and women, and were drawn using data from Supplementary Fig. 1 (panels a and b) and from Supplementary Table 7 (panels c and d)^11^.

Step 2 mandates the default application of either monotonic or linear tail constraints. These are applied so as “to regularise spline behaviour” and “to ensure plausible risk curve behaviour at low exposure levels”. However, examination of the Burden of Proof relative risk curves that describe the relationships of breast and colorectal cancer with unprocessed red meat, both with and without monotonic constraints, (Fig. 3)^11^ demonstrates significant risk inflationary effects. This is particularly evident at low to moderate intakes of unprocessed red meat. For colorectal cancer, the relative risks (95% confidence interval) associated with consumption of 25 g/day of unprocessed red meat prior to, and after application of the constraint, are 1.06 (0.9–1.23) and 1.28 (1.01–1.58), respectively. For breast cancer, the equivalent relative risks are 1.12 (0.94–1.35) and 1.26 (0.98–1.56), respectively. These two to four-fold increments in risk, arising through the application of monotonic constraints, do not appear reliable nor credible.

Step 4 entails the testing and correction for bias related to study design. Table 1 illustrates the contrast between GRADE methodology, which tests for bias across seven domains^37–39^, and that of the Burden of Proof study of the health effects associated with unprocessed red meat, which only tests for three possible sources of bias^11^. Furthermore, less stringent levels appear to be applied – Under adjustment for prognostic factors, no risk of bias is recorded if there is adjustment for age, sex, smoking and either income or level of education (Table 1). Hence, no adjustment for family history, alcohol, diabetes mellitus, dietary quantity (energy intake or body mass index) or quality (fibre, fruit, vegetables or ultraprocessed foods intake) is required, despite considerable evidence of their associations with all six outcomes.

**Table 1:** Comparison of the GRADE and the Burden of Proof study quality and risk of bias assessments for cohort studies evaluating the relationships between unprocessed red meat consumption and adverse outcomes.

	Source of Bias						
GRADE Questions & Responses	Selection	Assessment of exposure	Reverse causation	Adjustment for prognostic factors	Assessment of prognostic factors	Assessment of Outcome	Adequacy of follow-up
	Was selection of exposed and non‐exposed cohorts drawn from the same population?	Can we be confident in the assessment of exposure?	Can we be confident that the outcome of interest was not present at start of study?	Did the statistical analysis adjust for all variables that are associated with the outcome of interest?	Can we be confident in the assessment of the presence or absence of prognostic factors?	Can we be confident in the assessment of outcome?	Was the follow‐up of cohorts adequate?
Definitely yes	Selection for participation is not dependent on exposure level.	Participants complete a dietary measure at least every 5 years. The dietary measure has undergone validation against a weighted food record (correlation coefficient >0.4.)	Fatal outcomes. For non-fatal outcomes, there is an effort to exclude participants with the outcome of interest at baseline, with external validation of self-reports.	Adjusted for age, sex, smoking, socioeconomic status, family history, aspirin use, diabetes, alcohol, weight or BMI and physical activity.	Typically, prognostic factors are self‐reported by participants. This is considered acceptable.	All‐cause mortality based on a government registry. National or local registries or medical records with review by a study physician or study staff	At least 90% retention for the duration of the study.
Probably yes	Methods for recruitment are not adequately described so as to be able to determine whether recruitment into the study was dependent on intake of red meat	Participants complete a dietary measure at least every 6–8 years. The validation was not against a weighted food record, or the correlation coefficient is not reported.	The authors have made an effort to exclude participants with the outcome of interest at baseline. However, the outcome is self‐reported and there is no external validation reported.	Adjusted for age, sex, smoking, family history, and diabetes.	The study does not report how prognostic variables were measured.	Active follow-up or self‐report with external validation by medical records.	80 to 89% retention for the duration of the study with loss to follow‐up unlikely to be related to outcomes.
Probably no	–	Participants complete a dietary measure at least every 9–10 years. There is no report on the validity of the dietary measure, or the correlation coefficient <0.40.	–	Adjusted for age, sex, and smoking.	The study makes assumptions regarding various prognostic factors.	The authors do not specify how outcomes were measured, or medical records without review by study physician or study staff.	80 to 89% retention for the duration of the study with loss to follow‐up likely to be related to outcomes, or loss to follow‐up is not reported.
Definitely no	Studies that compare vegetarian and non‐vegetarian populations but draw vegetarians from a different cohort.	Participants complete a dietary measure only at baseline or less than every 10 years. The dietary measure has not undergone any validation.	No effort is made to exclude participants with the outcome of interest at baseline.	The study does not adjust for any prognostic variables relevant to the outcome or does not adjust for age, sex, or smoking.	–	Self‐report with no external validation.	Less than 80% follow‐up.

Burden of Proof Score	Selection	Assessment of exposure	Reverse causation	Adjustment for prognostic factors	Assessment of prognostic factors	Assessment of Outcome	Adequacy of follow-up
0	Source of bias not assessed	Multiple prospective objective measurements of red meat intake	Source of bias not assessed	Relative risk estimation analysis adjusted for age, sex, smoking and either income or education.	Source of bias not assessed	Objective report of outcome.	Source of bias not assessed
1		Multiple prospective self-reports of red meat intake, or Single baseline objective measurement of red meat intake		Adjusted for age, sex and smoking.		Self-report of outcome	
2		Single baseline self-report of red meat intake		Adjusted for age, and sex.		–	

It is noteworthy that, even with this limited testing for bias across only three domains, the mean quality score across all cohorts and outcomes was 2.8 (quality score range; 0 [least bias] to 5 [most bias])^11^. This high average score resulted from the majority of studies being judged to be at risk for bias because of use of a single self-report of red meat consumption, and only moderate adjustment for confounders. However, despite risks for bias being present in most studies, no bias covariates were identified as statistically relevant for inclusion in any of the six spline models^11^. This is an acknowledged limitation of the Burden of Proof methodology^12^ – the covariates cannot fully capture and correct for bias if all, or even a large majority, of the input studies are biased. It is of considerable concern that the end result is that no correction for bias related to study design was applied to the meta-regressions evaluating the relationships between unprocessed red meat consumption and health outcomes^11^.

Finally, it is not clear how the one to five star ratings of certainty (step 7) will be utilised in future GBD risk factor studies. The conclusion of the Burden of Proof study of the health effects associated with red meat consumption was that there was either no or only very weak evidence that unprocessed red meat is associated with any increased risk – only one or two stars were awarded. Lescinsky and colleagues concluded that this level of evidence is insufficient to make any strong or conclusive recommendations. However, it appears that the GBD collaborators do intend to include such weak, uncertain and statistically insignificant evidence in future GBD estimates of deaths and DALYs - they recently commented that their “preliminary estimates for GBD 2021 remain on the order of several hundred thousand deaths attributable to red meat consumption”^43^.

## Post publication processes – delayed or non-corrections of errors and limitations

Medical and scientific journals recognise the importance of post-publication commentary on published research as necessary to advancing scientific discourse. Hence, post-publication letters, commentaries and matters arising articles involving challenges or clarifications of the published work are generally welcomed. Once important errors are identified and confirmed, it is of considerable importance that appropriate corrections are rapidly published. Indeed, immediate correction of all errors of fact is mandatory according to The Lancet’s own guidelines, the Committee on Publication Ethics, and the International Committee of Medical Journal Editors^44–46^. It is of concern that, despite limitations and errors, being publically acknowledged by the EAT-Lancet and GBD authors, respectively, no corrections have been applied to the published papers, and the estimates remain unchanged on their websites^47,48^.

As a consequence these reports continue to be extensively cited, and many research groups continue to use the uncorrected estimates of risk and optimal intakes of the GBD 2019 Risk Factors Study and the EAT-Lancet planetary health diet, in their modelling studies^5–7,49–53^.

Fadnes and colleagues estimated that changing from a typical Western diet, which they (incorrectly) claimed included 100 g/day and 50 g/day of unprocessed red meat and processed meats, respectively, to a diet which totally excludes these foods, would increase life expectancy by at least three years for both men and women^5^. Romanello and colleagues in the 2022 report of the Lancet Countdown on health and climate change reported that approximately 800,000 and 600,000 annual deaths globally were caused by diets high in red meat and processed meat respectively^6^.

The same two publications also report deleterious effects to be associated with dairy consumption in excess of one helping each day. The online tool of Fadnes and colleagues (the Food4HealthyLife calculator) indicates that reduction of dairy or milk intake from 800 mls/day to 200 mls/day increases life expectancy by one year in young adults^5,53^. Romanello and colleagues identify 0–250 ml/day as the optimal intake for milk and dairy, and state that intakes above 250 ml/day contribute to overweight and obesity, and thereby result in approximately 600,000 cancer, cardiovascular or diabetic deaths annually^6^. Both publications appear to ignore the wealth of evidence that two or more daily helpings of full fat dairy (500–900 ml/day) provides protection against overweight, obesity and diabetes mellitus^54^, colorectal and breast cancer^55^, cardiovascular events and total mortality^56,57^.

Happily, recent reports from the Food and Agriculture Organisation of the United Nations and the World Health Organisation have recognised and highlighted the weaknesses and errors in these two reports^20,21^. However other agencies continue to be influenced.

The Livewell diet, which is the healthy sustainable diet recommended by the World Wildlife Fund includes even less animal-source foods than does the Eat-Lancet’s planetary health diet (meat; 34 g/day *versus* 43 g/day, eggs; 7 g/day *versus* 13 g/day, seafood; 40 g/day *versus* 28 g/day and dairy 192 g/day *versus* 250 g/day)^3,58^. Given recent reports of sizable micronutrient shortfalls pertaining to the Eat-Lancet’s planetary health diet^24–27^, the statement in the executive summary of the report that, “Livewell Plates are representative diets that meet national nutritional requirements,” does not appear correct.

The Nordic Nutrition Recommendations (NNR) 2023 recently ranked diets high in processed meat, and diets high in unprocessed red meat, as the second and fourth highest dietary risk factors, respectively, for mortality and DALYs, in the Nordic and Baltic countries^59^. It is cause for considerable concern that the evidence for this ranking is unpublished. The paper of Clarsen and colleagues^60^ was commissioned by the NNR Committee, but has been “in press” since March 2023 – it’s estimates of the burden of dietary risk factors in the Nordic and Baltic countries between 1990 and 2021, are based on data from the also unpublished Global Burden of Diseases, Injuries, and Risk Factors Study 2021.

## Conclusions

Science is the best method we have of coming to an impartial knowledge about the world^61^. In recent years there have been many calls for greater rigor, reproducibility and transparency across all the sciences^61–65^. In 2021 Brown and colleagues commented “Nutritional epidemiology can, and must, do better by pursuing greater scientific rigor, academic honesty, and intellectual integrity”^65^. Hence, in the conduct of systematic reviews of dietary factors, in the estimation of global health estimates, and in the use of these metrics in policy decisions and dietary guidelines, nutritional epidemiology must follow similar or analogous regulations and standards as all other scientific endeavours. In determining the optimal intakes of foods, the impacts of both nutritional deficiencies and excesses must be considered. Differences in micronutrient bioavailability from different food sources must also be recognised. PRISMA-compliant reports of all systematic reviews, and GATHER-compliant reports of all global health estimates must be published. Assessments of bias and certainty in nutrition science must be of a similar standard as those in all other health-related fields. Curve smoothing techniques cannot be allowed to inflate or create risk. Confirmed substantial errors must be immediately corrected in all paper and on-line publications, and also on institutional websites. Given the huge influence of global health estimates from the GBD Risk Factor Collaborators, and from the EAT-Lancet Commission on Food, Planet, Health, it is of even greater importance that the metrics and recommendations from these groups are rigorously and transparently evidence-based.
